# Interplay of Frailty, Intrinsic Capacity, and Cardiorespiratory Fitness in Older Indian Adults: Insights From a Cross-Sectional Study

**DOI:** 10.7759/cureus.107740

**Published:** 2026-04-26

**Authors:** Bhrigu Jain, Avinash Chakrawarty, Prasun Chatterjee, Aparajit Ballav Dey, Maroof Khan

**Affiliations:** 1 Department of Geriatric Medicine, All India Institute of Medical Sciences, New Delhi, New Delhi, IND; 2 Department of Geriatric Medicine, Venu Charitable Society, New Delhi, IND; 3 Department of Biostatistics, All India Institute of Medical Sciences, New Delhi, New Delhi, IND

**Keywords:** cardiorespiratory fitness, frailty, intrinsic capacity, older adults, vo₂max

## Abstract

Background

Frailty, intrinsic capacity (IC), and cardiorespiratory fitness each reflect physiological reserve in aging, yet integrated data combining standardized cardiopulmonary exercise testing (CPET) with the World Health Organization (WHO) Integrated Care for Older People (ICOPE) framework and the Fried phenotype are scarce, particularly in South Asian older adults, where the burden of chronic disease may accelerate functional decline.

Methods

This cross-sectional study included 130 healthcare-seeking adults aged ≥65 years attending a tertiary geriatric outpatient clinic in India. Frailty was assessed using the Fried phenotype, intrinsic capacity using the World Health Organization Integrated Care for Older People framework across five domains, and cardiorespiratory fitness using CPET to determine maximal oxygen uptake (VO₂max). Associations between IC, frailty, and VO₂max were examined using univariate and multivariable linear regression analyses.

Results

Frailty prevalence was 73.1% (95/130 participants). Frail participants demonstrated significantly lower skeletal muscle mass, poorer functional performance, and reduced cardiorespiratory fitness compared with non-frail individuals. Higher IC impairment scores were associated with older age, poorer anthropometric measures, reduced physical performance, and lower absolute VO₂max (all p < 0.05). In univariate analyses, several variables, including age, skeletal muscle mass, handgrip strength, gait speed, physical activity, and IC domains, were associated with VO₂max. In a prespecified multivariable regression model adjusting for age, sex, and frailty status, IC total score remained independently associated with lower absolute VO₂max (β ≈ -31 mL/min per point, p ≈ 0.03). Frailty demonstrated a borderline association but did not retain statistical significance after adjustment.

Conclusions

Impairment of intrinsic capacity was independently associated with lower cardiorespiratory fitness in older adults, independent of age, sex, and frailty status. These cross-sectional findings are hypothesis-generating, and prospective studies are required to determine whether intrinsic capacity precedes decline in aerobic fitness and frailty. Integrating intrinsic capacity assessment with objective measures of aerobic fitness may improve early identification of vulnerable older adults and inform preventive geriatric care strategies.

## Introduction

The global population is undergoing rapid aging, with the number of individuals aged 60 years and older projected to double by 2050, exceeding two billion worldwide [1]. In India, this demographic transition is particularly pronounced, driven by improved life expectancy and declining fertility rates, resulting in an estimated 340 million older adults by 2050 [2].

This demographic shift is accompanied by an aging population with progressive multisystem declines, including musculoskeletal atrophy, cardiovascular inefficiency, and neurological impairments, which reduce physiological reserves and increase vulnerability to external stressors [3]. These age-related changes manifest as diminished physical performance, cognitive deceleration, and metabolic imbalances, predisposing individuals to adverse outcomes such as falls, hospitalizations, loss of independence, and premature mortality [4].

Moreover, these declines are not solely chronological but are often exacerbated by modifiable factors like sedentary lifestyles, chronic comorbidities, and socioeconomic disparities. At the core of this functional erosion lie three interrelated constructs: frailty, intrinsic capacity (IC), and peak oxygen uptake (VO₂max). Frailty represents a multidimensional syndrome of heightened vulnerability to external stressors arising from accumulated deficits across physiological systems, evolving from robust to pre-frail and frail states [3,4]. IC, a paradigm put forward by the World Health Organization (WHO), integrates an individual's overall functional abilities across five key domains: cognition, sensory function, vitality (encompassing nutrition), locomotion, and psychological health, serving as a measure of the body's inherent reserve to meet daily demands [5,6]. VO₂max, the benchmark for cardiorespiratory fitness, quantifies the maximum rate of oxygen transport and utilization during intense exercise, measuring the efficiency of cardiovascular, pulmonary, and muscular systems [7].

With advancing age, these elements exhibit nonlinear deterioration: frailty prevalence increases, doubling every five to seven years after age 65; IC diminishes by approximately 1-2% annually in locomotion and vitality domains; and VO₂max declines by 5-10% per decade, with acceleration beyond age 70 years [3,8,9]. This triad engages in a vicious cycle, wherein diminished VO₂max promotes deconditioning and frailty progression, while IC impairments exacerbate both, culminating in a trajectory toward disability and dependency. Prognostically, frailty confers a two to three-fold increase in mortality risk [10], IC deficits predict dependency and healthcare utilization [11], and VO₂max thresholds below 18 ml/kg/min herald cardiovascular events and all-cause mortality [9]. Thus, these constructs, with their robust predictive utility, facilitate early identification and intervention in geriatric practice. In this study, we operationalized frailty using the Fried phenotype, intrinsic capacity using the WHO-Integrated Care for Older People (ICOPE) framework, and cardiorespiratory fitness was ascertained through cardiopulmonary exercise testing (CPET), following guidelines from the American Thoracic Society (ATS) and American College of Chest Physicians (ACCP) [12].

Frailty and diminished intrinsic capacity frequently coexist in older adults, with substantial overlap observed in 30-55% of community-dwelling individuals, particularly in advanced age groups [13]. This interconnection is underpinned by shared pathophysiological pathways, including chronic low-grade inflammation (elevated interleukin-6 and C-reactive protein), sarcopenia, and mitochondrial dysfunction, which collectively impair cellular energy production, muscle oxidative capacity, and systemic tissue perfusion. Reduced cardiorespiratory fitness functions as a central physiological intermediary of this declining reserve. Frail or IC-impaired individuals typically exhibit lower aerobic capacity, largely attributable to mitochondrial bioenergetic failure and impaired peripheral oxygen extraction. Cross-sectional investigations further demonstrate that intrinsic capacity deficits independently explain a clinically meaningful proportion of VO₂max variance beyond frailty status [14], while longitudinal evidence reveals that the combined presence of both conditions synergistically elevates the risk of functional disability and loss of independence [15]. 

Despite these insights, significant knowledge gaps persist. The majority of research is derived from Western populations, under-representing South Asian cohorts where high burdens of diabetes, hypertension, and malnutrition may hasten physiological decline. Furthermore, integrated measurements combining standardized CPET with IC and frailty assessments are infrequently employed in resource-limited contexts, hindering the development of context-specific interventions. Against this background, we conducted the present cross-sectional study in older adults attending a tertiary geriatric clinic in India, applying established frameworks for each construct (Fried phenotype for frailty, WHO-ICOPE for IC, and ATS/ACCP guidance for CPET). The primary objective was to determine whether intrinsic capacity impairment is independently associated with absolute VO₂max after adjustment for age, sex, and frailty status. Secondary objectives were (a) to describe differences in anthropometric, functional, and CPET-derived parameters between frail and non-frail participants, and (b) to characterize the associations of individual IC domains with absolute VO₂max. We hypothesized that greater IC impairment would be associated with lower aerobic capacity and that IC would contribute information beyond the Fried phenotype in accounting for variation in VO₂max. The findings are intended to inform integrated geriatric care strategies for low- and middle-income settings.

## Materials and methods

Study design and sampling

This cross-sectional study enrolled healthcare-seeking older adults aged ≥65 years presenting to the Outpatient Department of Geriatric Medicine at the All India Institute of Medical Sciences (AIIMS), Delhi, India, between January and June 2019. The sampling frame comprised consecutive attendees to the geriatric outpatient department (OPD) on designated research days, aligned with the researcher's availability and focused on patients from a single assigned OPD room to facilitate logistical feasibility in a high-volume clinical setting. In the absence of prior data on the interrelationships among intrinsic capacity, frailty, and peak oxygen uptake in this population, a convenience sampling approach was employed to detect clinically relevant associations. From an initial pool of 200 eligible patients assessed during the study period, a total of 130 participants (90 males and 40 females) were recruited following application of predefined inclusion and exclusion criteria.

Eligibility criteria

Inclusion criteria encompassed adults aged ≥65 years who were willing and able to provide informed consent, attend study assessments, and undergo CPET safely, without absolute contraindications such as recent acute myocardial infarction, unstable angina, uncontrolled cardiac arrhythmias, symptomatic severe aortic stenosis, decompensated heart failure, uncontrolled asthma or COPD, resting room air oxygen saturation ≤85%, acute noncardiopulmonary disorders (e.g., active infection, acute kidney or liver injury or decompensation) that could be exacerbated by exercise, or mental impairment precluding cooperation. Exclusion criteria included severe cognitive impairment, dependence on mobility aids for ambulation, orthopedic impairments severely limiting exercise, or unwillingness to participate. The study protocol received ethical approval from the AIIMS Institutional Ethics Committee (IECPG-365/28/09/2017), and all participants provided written informed consent prior to enrollment.

Comprehensive baseline assessment

Participants underwent comprehensive evaluations, including a detailed physical examination, collection of demographic data on socioeconomic status (low or high) and various covariates such as educational status as low (primary, secondary) or high (graduate/post-graduate), physical activity (assessed by a single screening question from the WHO-ICOPE toolkit: “Do you perform any regular physical activity or exercise, such as brisk walking, yoga, gardening, cycling, or gym activities, at least three to four times per week for 20-30 minutes or more?”; “yes” classified as physically active).

Comorbidities were assessed through self-reports and corroborated by medical records (e.g., diabetes mellitus (DM), hypertension (HTN), coronary artery disease (CAD), chronic obstructive pulmonary disease (COPD), dyslipidemia, hypothyroidism). Under geriatric assessment, history of falls (self-reported any unintentional fall to the ground in the last year), urinary incontinence (self-reported any involuntary leakage of urine in the past six months), and polypharmacy (defined as taking ≥5 prescription medications daily, ascertained from medication review) were recorded. Any impairment in carrying out basic activities of daily living was recorded. 

Anthropometric measurements were obtained using calibrated equipment. Weight and height were measured for body mass index (BMI) calculation, and bioelectrical impedance analysis (BIA) was used to estimate skeletal muscle mass (SMM) and appendicular skeletal muscle index (ASMI). BIA device BCA-2A, provided by Tsinghua Tongfang Co., Ltd., Beijing, China, was used. It was a segmental multi-frequency machine with five test frequencies (5, 50, 100, 250, and 500 kHz). Patients were measured in the morning with an empty bladder, wearing light clothing, and standing barefoot for at least one minute on the platform, with hands and feet in contact with electrodes, to complete the electrical circuit. A minuscule current of 500 μA was passed through this completed circuit, and readings were taken. All metallic ornaments, coins, mobile phones, cashless cards, watches, and glasses were removed before the testing. ASMI was calculated by dividing the appendicular skeletal muscle mass value by height in m². 

Assessment of frailty

Frailty was assessed using the Fried phenotype model, which has been validated and widely applied in Indian populations, with studies confirming its reliability and predictive validity for adverse outcomes in this demographic [16]. As an open-access tool described in the public domain, no specific permissions are required for its use in research. This involved scoring five criteria: (1) shrinking, defined as unintentional weight loss exceeding 4.5 kg in the past year or BMI <18.5 kg/m²; (2) weakness, measured as dominant handgrip strength <20 kg for women or <30 kg for men, averaged over three trials with a Jamar dynamometer; (3) exhaustion, self-reported as occurring ≥3 days per week via the Center for Epidemiologic Studies Depression (CES-D) subscale, which has been culturally adapted and validated for use in Indian and Asian populations [17,18]; (4) slowness, quantified as gait speed <0.8 m/s over a 4-meter course, using the fastest of two trials; (5) low physical activity, assessed as energy expenditure <383 kcal/week for men or <270 kcal/week for women using the Minnesota Leisure Time Physical Activity Questionnaire, a tool originally developed in Western contexts but applied in Indian and Asian studies with adaptations for local activities, such as household tasks and cultural practices [19,20]. A total score of ≥3 out of 5 denoted frailty. 

Assessment of intrinsic capacity

IC was measured according to WHO guidance as the composite sum of five domains: sensory, cognition, locomotion, vitality, and psychological. Cognition was assessed using an ad hoc Hindi-translated Mini-Cog after obtaining permission for use, with a score ≤2 indicating cognitive impairment [21]. Locomotion was assessed using the short physical performance battery. A score ≤9 indicating impairment [22]. Vitality was assessed using an ad hoc Hindi-translated DETERMINE checklist, with a score ≥3 suggesting risk of malnourishment [23]. The sensory domain was assessed by self-reported vision and hearing ability. Patients were asked if they had experienced any decline in vision and hearing in daily life, even while using their usual glasses and/or hearing aids (if applicable). Those with intact vision and hearing were given a score of 0, and those with any impairment (in vision or hearing or both) were given a score of 1. Psychological capacity was assessed using an ad-hoc Hindi-translated 5-item Geriatric Depression Scale (GDS-5), with a score of ≥2 indicating impairment [24].

If any domain was impaired, it was given a score of “1”, otherwise “0”. The composite score for IC was calculated by summing the scores across all five domains. The composite score ranged from 0 to 5, with a higher score indicating lower intrinsic capacity. Impaired IC was defined as having deficits in one or more domains. Furthermore, if any screening test in any domain was found positive, patients underwent a detailed assessment for further management. Only the initial screening tests were used to compute IC per WHO guidance and were included in the final analysis. Apart from Mini-Cog, all the tools used for the evaluation of IC were available in open access, so no special permissions were needed for the rest.

Assessment of cardiopulmonary testing

Cardiorespiratory fitness indices, including VO₂max, were measured through cardiopulmonary exercise testing, which provides a global assessment of the exercise responses as per ATS/ACCP guidelines [12]. Prior to each session, daily calibration of the flow meters and gas analyzers was performed, and proper safety measures were ensured, including the availability of a resuscitation cart. Predicted values were calculated using Wasserman/Hansen equations based on anthropometric data [25]. Pre-exercise pulmonary function testing was conducted in a standing position to rule out any pulmonary pathology that could alter results. Participants were seated on the COSMED cycle ergometer after seat-height adjustment and instructed to grip the handlebars; an appropriately sized face mask was fitted, along with ECG electrodes and a pulse oximetry probe, before the symptom-limited incremental test began.

Breath-by-breath gas-exchange analysis provided real-time data on peak oxygen uptake (VO₂max; maximum rate of oxygen consumption during exercise, reported absolute in ml/min and weight-normalised and skeletal muscle mass normalized in ml/min/Kg), carbon dioxide output (VCO₂; volume of CO₂ produced per minute), minute ventilation (VE; total volume of air breathed per minute), anaerobic threshold (AT; exercise intensity at which anaerobic metabolism supplements aerobic energy production, identified by the V-slope method), ventilatory equivalents (VE/VO₂ and VE/VCO₂; markers of breathing efficiency relative to oxygen consumption and carbon dioxide production) and respiratory exchange ratio (RER = VCO₂/VO₂; indicator of fuel substrate utilisation and maximal effort).

Each participant underwent a progressively incremental exercise protocol with a ramp design: a one-minute rest period followed by a one-minute warmup phase at 0 Watts with pedaling cadence maintained below 30 revolutions per minute (RPM), then the test phase starting at an initial load of 20 Watts with 15-Watt/minute increments, during which patients were encouraged to maintain RPM at 55-60, with no talking permitted and discomfort signaled by head shake or hand raise. Continuous monitoring of 12-lead electrocardiogram (ECG), blood pressure (BP; measured at two-minute intervals), and peripheral oxygen saturation (SpO₂) ensured participant safety throughout the test, with immediate termination if adverse events occurred. Leading yes/no questions assessed perceived dyspnea and leg pain via the modified Borg scale during exercise [26]. The test concluded upon achieving any of the following outcomes: maximum heart rate >90% of predicted value, RER >1.15, Borg scale rating ≥18 for dyspnea/fatigue; or ECG changes, abnormal BP response, significant desaturation, or patient request. Observed values were compared with predicted values to determine whether peak VO₂max was reached, and the anaerobic threshold was calculated using software and cross-verified visually via the V-slope method.

Statistical analysis

All statistical analyses were performed using IBM SPSS Statistics for Windows, version 26.0 (IBM Corp., Armonk, New York). Continuous variables were expressed as mean ± standard deviation (SD) when normally distributed and as median with interquartile range (IQR) when non-normally distributed; categorical variables were summarized as frequencies and percentages. Normality was assessed using the Shapiro-Wilk test and confirmed by visual inspection of histograms and Q-Q plots. Between-group comparisons according to frailty status were conducted using independent-samples t-tests or Mann-Whitney U tests for continuous variables and the χ² test or Fisher’s exact test (when expected cell counts were <5) for categorical variables, as appropriate. Univariate associations between clinical variables and continuous outcomes were assessed using simple linear regression. Two principal continuous outcomes were examined: (1) intrinsic capacity total score and (2) absolute peak oxygen uptake (VO₂max, mL/min). Regression coefficients (β), 95% confidence intervals (CI), and p-values were reported. For the primary analysis evaluating determinants of cardiorespiratory fitness, a prespecified multivariable linear regression model was constructed with absolute VO₂max (mL/min) as the dependent variable. We chose absolute VO₂max as the primary outcome because older adults frequently show sarcopenic obesity, and adipose tissue contributes little to oxygen consumption. Hence, ratio-normalization to total body mass can attenuate true physiological differences. Age and sex were included a priori due to their established physiological influence on aerobic capacity. Intrinsic capacity total score was entered as the primary exposure variable of interest. Frailty status was additionally included to account for conceptual and clinical overlap with intrinsic capacity. No automated forward or backward variable selection procedures were employed; covariates were selected based on biological plausibility and theoretical relevance rather than purely statistical criteria. Model performance was evaluated using the coefficient of determination (R²), adjusted R², and the overall F-statistic. Multicollinearity was assessed using variance inflation factors (VIF), with values <5 considered acceptable. Model assumptions were evaluated through inspection of residual plots to confirm linearity and homoscedasticity, and Cook’s distance was examined to identify potentially influential observations. With 130 complete-case participants and four predictors in the primary multivariable model (age, sex, frailty status, intrinsic capacity), the ratio of approximately 32 observations per predictor exceeds the conventional minimum of 10-15 per predictor for linear regression, supporting model stability. All analyses were conducted using complete-case data; no imputation was required. Univariate analyses were exploratory and hypothesis-generating; no adjustment for multiple testing was applied, and the possibility of type I error inflation is acknowledged. Primary inference rests on the prespecified multivariable model. All statistical tests were two-tailed, and a p-value <0.05 was considered statistically significant. Figures were generated after data export for graphical presentation. The primary figure illustrates the relationship between intrinsic capacity and absolute VO₂max, and the adjusted effects from the multivariable model are presented using regression coefficient plots.

## Results

Participant characteristics and frailty associations

A total of 130 participants were included in the analysis, comprising 35 non-frail and 95 frail individuals. The overall mean age was 70.8 ± 4.9 years, with 31% female representation. Frailty prevalence was 73.1%. Frail participants were significantly older than non-frail participants (72.2 vs. 67.2 years, p < 0.001), while sex distribution, socioeconomic status, and education level did not differ between groups (Table 1). Because frail participants were older, several of the unadjusted differences below may partly reflect age rather than frailty alone; this motivated the prespecified multivariable model. Frail participants showed poorer body composition, with lower median body weight (60.0 vs. 66.0 kg), skeletal muscle mass, BMI, and ASMI (Table 1). Cardiorespiratory fitness was also compromised: absolute VO₂max was approximately 23% lower in the frail group (median 925 vs. 1199 ml/min), VO₂max/SMM was lower, and oxygen pulse was reduced. VO₂max/kg and the anaerobic threshold did not differ between groups, while VE/VCO₂ was higher among frail individuals (35.6 vs. 32.4), indicating greater ventilatory inefficiency. Comorbidity profiles were broadly similar between groups. Functional impairment was markedly more common among frail participants, with handgrip strength approximately 9 kg lower (20 vs. 29 kg), gait speed 0.14 m/s slower (0.68 vs. 0.82 m/s), and a higher frequency of falls, low physical activity, and impaired ADLs. Locomotion and vitality impairments were substantially more prevalent among frail participants (61% vs. 11% and 62% vs. 26%, respectively), and the IC total score was higher (median 3 vs. 1). Higher IC total scores denote greater domain impairment and a lower Intrinsic capacity.

**Table 1:** Baseline characteristics of participants according to frailty status. Baseline demographic, anthropometric, clinical, and functional characteristics of study participants stratified by frailty status according to the Fried phenotype. Continuous variables are presented as mean ± standard deviation (normally distributed) or median (interquartile range) (non-normally distributed); categorical variables are shown as n (%). Between-group comparisons were performed using the independent-samples t-test (t), Mann-Whitney U test (U), or chi-square test (χ²) as appropriate. Higher IC total scores indicate greater domain impairment and a lower IC. ASMI: appendicular skeletal muscle index; SMM: skeletal muscle mass; VO₂max: maximal oxygen uptake; AT: anaerobic threshold; O₂ pulse: oxygen pulse; VE/VCO₂: ventilatory equivalent for carbon dioxide; HR max: maximum heart rate; ADLs: activities of daily living; IC: intrinsic capacity.

Variable	Non-frail (n = 35)	Frail (n = 95)	P-value	Test statistic
Demographics				
Sex (female %)	11 (31.4%)	29 (30.5%)	0.946	χ² = 0.00
Age (years)	67.2 ± 2.7	72.2 ± 6.1	<0.001	t = 4.56
Socioeconomic status (low %)	12 (34.3%)	32 (33.7%)	0.789	χ² = 0.07
Education (low %)	19 (54.3%)	55 (57.9%)	0.679	χ² = 0.17
Anthropometric measures				
Weight (kg)	66.0 (60.0-69.5)	60.0 (52.5-65.0)	0.005	t = 2.85
Skeletal mass (kg)	30.8 (27.9-33.5)	28.5 (25.5-32.4)	0.023	t = 2.31
BMI (kg/m²)	25.2 ± 3.8	23.1 ± 3.8	0.012	t = 2.54
ASMI (kg/m²)	8.2 ± 0.8	7.5 ± 0.9	<0.001	t = 4.12
Cardiorespiratory fitness				
VO_2_ max (ml/min)	1199 (892-1345)	925 (771-1096)	0.002	U = 1056
VO_2_max/kg (ml/min/kg)	16.9 (14.3-20.3)	16.0 (13.4-18.5)	0.309	t = 1.02
VO_2_max/SMM (ml/min/kg)	36.2 (32.9-40.4)	33.0 (28.8-37.1)	0.046	t = 2.02
AT (ml/min)	861 (637-999)	754 (582-910)	0.140	U = 1423
O2 pulse (ml/beat)	9.3 (7.1-10.0)	7.1 (5.6-8.8)	0.005	U = 1098
VE/VCO_2_ ratio	32.4 ± 3.1	35.6 ± 4.7	<0.001	t = 3.89
HR max (beats/min)	129 ± 14	129 ± 15	0.972	t = 0.03
Comorbidities				
Diabetes mellitus	7 (20.0%)	33 (34.7%)	0.127	χ² = 2.34
Hypertension	22 (62.9%)	61 (64.2%)	0.912	χ² = 0.01
Dyslipidemia	4 (11.4%)	16 (16.8%)	0.457	χ² = 0.55
Hypothyroidism	7 (20.6%)	12 (12.6%)	0.153	χ² = 2.67
Functional measures and geriatric syndromes				
Hand grip (kg)	29 (26-32)	20 (14-24)	<0.001	U = 512
Gait speed (m/s)	0.82 (0.77-0.89)	0.68 (0.63-0.73)	<0.001	U = 623
Falls	7 (20.0%)	40 (42.1%)	0.023	χ² = 5.18
Urinary incontinence	9 (25.7%)	20 (21.1%)	0.568	χ² = 0.33
Physical activity (active %)	34 (97.1%)	72 (75.8%)	0.012	χ² = 6.34
Polypharmacy	10 (28.6%)	36 (37.9%)	0.346	χ² = 0.89
Impaired ADLs	5 (14.3%)	37 (38.9%)	0.001	χ² = 7.12
Intrinsic capacity domains				
Sensory IC (impaired %)	28 (80.0%)	72 (75.8%)	0.568	χ² = 0.33
Cognition IC (impaired %)	7 (20.0%)	27 (28.4%)	0.346	χ² = 0.89
Locomotion IC (impaired %)	4 (11.4%)	58 (61.1%)	<0.001	χ² = 24.56
Vitality IC (impaired %)	9 (25.7%)	59 (62.1%)	<0.001	χ² = 13.89
Psychological IC (impaired %)	12 (34.3%)	45 (47.4%)	0.123	χ² = 2.38
Intrinsic capacity total score	1 (1-2)	3 (2-4)	0.001	U = 789

Associations of intrinsic capacity with clinical and functional measures

The median number of impaired IC domains was 2 (IQR 1-3), and 75% of participants had at least one domain deficit. Univariate associations between IC total score and participant characteristics are shown in Table 2. Higher IC impairment was associated with older age, female sex, lower socioeconomic status and education, and poorer anthropometric measures (lower weight, skeletal muscle mass, BMI, and ASMI). Among cardiorespiratory fitness indices, greater IC impairment was associated with lower absolute VO₂max, lower anaerobic threshold, reduced oxygen pulse, and higher VE/VCO₂, whereas VO₂max/kg and VO₂max/SMM were not associated. IC impairment was also strongly associated with frailty status and with all assessed functional measures (handgrip strength, gait speed, physical activity, history of falls, and impaired ADLs). No significant associations were observed with individual comorbidities, urinary incontinence, or polypharmacy.

**Table 2:** Univariate associations of intrinsic capacity total score. Univariate linear regression analyses examining associations between participant characteristics and the intrinsic capacity (IC) total score. β coefficients represent the change in IC impairment score per unit increase in the predictor variable (or per IC point for the outcome). Higher IC scores indicate greater impairment across domains. VO₂max: maximal oxygen uptake; SMM: skeletal muscle mass; ASMI: appendicular skeletal muscle index; IC: intrinsic capacity; ADLs: activities of daily living. Coding of categorical variables: sex (0 = male, 1 = female); Fried phenotype (0 = non-frail, 1 = frail); socioeconomic status (0 = high, 1 = low); education (0 = higher education, 1 = low education); physical activity (0 = inactive, 1 = active); history of falls (0 = no, 1 = yes); urinary incontinence (0 = no, 1 = yes); polypharmacy (0 = <5 medications, 1 = ≥5 medications); impaired ADLs (0 = none, 1 = ≥1 impairment).

Variable	β (per IC point)	95% CI	P-value
Anthropometric measures and demographics			
Weight (kg)	-0.053	-0.072 to -0.034	<0.001
Skeletal mass (kg)	-0.116	-0.159 to -0.073	<0.001
BMI (kg/m²)	-0.115	-0.172 to -0.059	<0.001
ASMI (kg/m²)	-0.668	-0.879 to -0.457	<0.001
Sex (0 = male, 1 = female)	0.514	0.018 to 1.010	0.043
Age (years)	0.051	0.012 to 0.089	0.011
Socioeconomic status	-0.930	-1.333 to -0.527	<0.001
Education	-0.401	-0.679 to -0.123	0.005
Cardiorespiratory fitness indices			
VO₂ max (ml/min)	-0.00186	-0.00269 to -0.00103	<0.001
VO₂ max/kg (ml/min/kg)	-0.0216	-0.0807 to 0.0375	0.471
VO₂ max/SMM (ml/min/kg)	-0.0195	-0.0535 to 0.0146	0.260
Anaerobic threshold (ml/min)	-0.00159	-0.00251 to -0.00067	0.001
O₂ pulse (ml/beat)	-0.192	-0.298 to -0.087	<0.001
VE/VCO₂ ratio	0.085	0.036 to 0.134	0.001
HR max (beats/min)	-0.0135	-0.029 to 0.002	0.091
Comorbidities and frailty			
Fried phenotype	1.033	0.540 to 1.526	<0.001
Diabetes mellitus	-0.353	-0.853 to 0.148	0.166
Hypertension	-0.398	-0.878 to 0.081	0.103
Dyslipidemia	0.095	-0.550 to 0.741	0.770
Hypothyroidism	-0.276	-0.906 to 0.355	0.389
Functional measures and geriatric syndromes			
Hand grip strength (kg)	-0.095	-0.123 to -0.066	<0.001
Gait speed (m/s)	-5.319	-7.447 to -3.191	<0.001
History of falls	0.765	0.299 to 1.230	0.001
Urinary incontinence	0.372	-0.183 to 0.928	0.187
Physical activity	-1.417	-1.964 to -0.871	<0.001
Polypharmacy	0.249	-0.235 to 0.734	0.311
Impaired ADLs	1.206	0.755 to 1.657	<0.001

Univariate predictors of absolute VO₂max

Univariate predictors of absolute VO₂max are shown in Table 3. Greater body weight, skeletal muscle mass, and ASMI were positively associated with VO₂max, while female sex and older age were associated with lower values. Frailty, impaired ADLs, and each of the cognition, locomotion, vitality, and psychological IC domains were associated with lower VO₂max, whereas handgrip strength, gait speed, and physical activity were positively associated. Socioeconomic status, education, and individual comorbidities were not significantly associated. A higher IC total score was associated with reduced VO₂max (β = −71.67 per point).

**Table 3:** Univariate predictors of absolute maximal oxygen uptake. Univariate linear regression analyses assessing associations between demographic, anthropometric, clinical, functional, and intrinsic capacity variables and absolute maximal oxygen uptake (VO₂max). β coefficients represent the change in VO₂max (ml/min) per unit increase in the predictor variable. ASMI: appendicular skeletal muscle index; VO₂max: maximal oxygen uptake; IC: intrinsic capacity; ADLs: activities of daily living. Coding of categorical variables: sex (0 = male, 1 = female); Fried phenotype (0 = non-frail, 1 = frail); socioeconomic status (0 = high, 1 = low); education (0 = higher education, 1 = low education); physical activity (0 = inactive, 1 = active); history of falls (0 = no, 1 = yes); urinary incontinence (0 = no, 1 = yes); polypharmacy (0 = <5 medications, 1 = ≥5 medications); impaired ADLs (0 = none, 1 = ≥1 impairment); intrinsic capacity domains (0 = intact, 1 = impaired).

Predictor	β	95% CI	P-value
Anthropometric measures and demographics			
Weight (kg)	10.97	7.28 to 14.66	<0.001
Skeletal mass (kg)	33.74	26.58 to 40.89	<0.001
BMI (kg/m²)	11.61	-0.03 to 23.24	0.050
ASMI (kg/m²)	147.16	107.37 to 186.96	<0.001
Sex (0 = male, 1 = female)	-297.52	-381.80 to -213.23	<0.001
Age (years)	-9.92	-17.57 to -2.27	0.011
Socioeconomic status	53.35	-31.49 to 138.18	0.216
Education	19.42	-36.77 to 75.62	0.495
Comorbidities and frailty			
Fried phenotype	-166.56	-265.43 to -67.70	0.001
Diabetes mellitus	40.30	-58.50 to 139.10	0.421
Hypertension	52.29	-42.42 to 147.00	0.277
Dyslipidemia	21.54	-105.11 to 148.19	0.737
Hypothyroidism	89.54	-33.69 to 212.76	0.153
Functional measures and geriatric syndromes			
Hand grip strength (kg)	22.01	16.80 to 27.22	<0.001
Gait speed (m/s)	1031.79	613.07 to 1450.51	<0.001
History of falls	-84.98	-178.97 to 9.01	0.076
Urinary incontinence	-62.66	-171.92 to 46.61	0.259
Physical activity	177.91	64.26 to 291.56	0.002
Polypharmacy	-13.93	-109.51 to 81.65	0.774
Impaired ADLs	-128.91	-224.03 to -33.79	0.008
Intrinsic capacity domains			
Sensory IC	14.72	-93.76 to 123.20	0.789
Cognition IC	-158.91	-259.15 to -58.66	0.002
Locomotion IC	-188.69	-274.07 to -103.32	<0.001
Vitality IC	-105.01	-194.68 to -15.34	0.022
Psychological IC	-104.23	-194.55 to -13.92	0.024
IC total score (0-5)	-71.67	-103.64 to -39.70	<0.001

**Figure 1:**
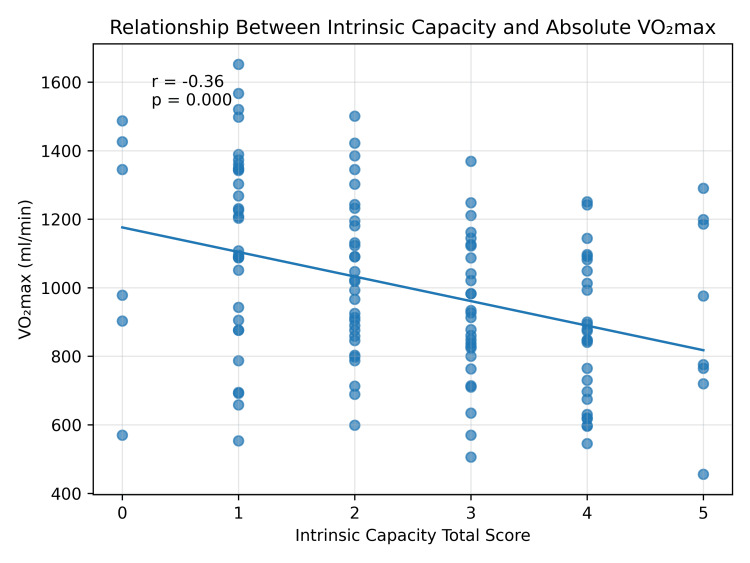
Relationship between intrinsic capacity impairment and absolute VO₂max. Association between intrinsic capacity (IC) total score and absolute maximal oxygen uptake (VO₂max). Each point represents an individual participant. The fitted regression line demonstrates a significant inverse relationship between increasing IC impairment and aerobic capacity, indicating that individuals with higher IC impairment scores exhibit lower VO₂max values. Correlation was assessed using Pearson correlation analysis (r = −0.36, p < 0.001).

Figure 1 illustrates a clear downward trend in absolute VO₂max with increasing IC impairment, with greater clustering of lower aerobic capacity values at higher IC scores.

Multivariable predictors of absolute VO₂max

Multivariable linear regression analysis was performed to evaluate independent determinants of absolute VO₂max (Table 4). A prespecified model, adjusting for age, sex, total intrinsic capacity score, and frailty status, was constructed based on clinical relevance and biological plausibility. The model explained 45.5% of the variance in VO₂max (R² = 0.455; adjusted R² = 0.437; p < 0.001). Age was independently associated with lower VO₂max (p = 0.0003), and female sex remained a strong independent determinant (p < 0.001). Importantly, the IC total score remained an independent negative predictor of absolute VO₂max (β = −31.00 per point increase, p = 0.032), indicating that greater cumulative impairment of intrinsic capacity is associated with reduced cardiorespiratory fitness beyond the effects of age and sex. Frailty demonstrated a borderline negative association (p = 0.096), suggesting partial overlap with IC but no independent statistical significance in the fully adjusted model.

**Table 4:** Multivariable linear regression model for absolute VO₂max. Multivariable linear regression model evaluating independent predictors of absolute maximal oxygen uptake (VO₂max). The final model included the intrinsic capacity (IC) total score, Fried frailty phenotype, age, and sex. β: unstandardized regression coefficient; SE: standard error; 95% CI: 95% confidence interval; VIF: variance inflation factor (assessed for multicollinearity); IC: intrinsic capacity; VO₂max: maximal oxygen uptake. Fried phenotype was coded as a binary variable (0 = non-frail, 1 = frail). Sex was coded as 0 = male and 1 = female.

Predictor	β (SE)	95% CI	P-value	VIF
Intercept	2127.27 (230.68)	1671.47-2583.07	<0.001	-
IC total score (0-5)	-31.00 (14.28)	-59.24-2.76	0.032	1.21
Fried phenotype (0 = non-frail, 1 = frail)	-73.81 (43.98)	-160.88-13.27	0.095	1.28
Age (years)	-12.68 (3.38)	-19.37-6.00	<0.001	1.30
Sex (0 = male, 1 = female)	-322.75 (39.92)	-401.78-243.73	<0.001	1.14

**Figure 2:**
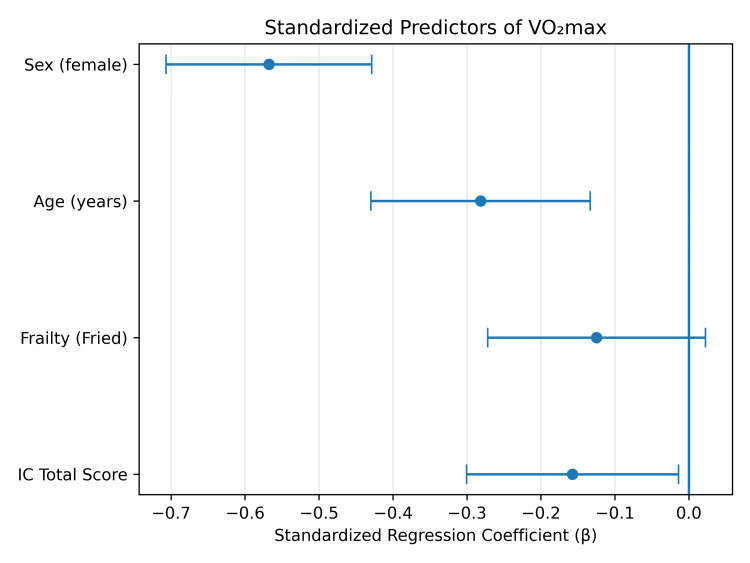
Standardized predictors of absolute VO₂max in the multivariable regression model. Standardized regression coefficients from the multivariable linear regression model examining predictors of absolute maximal oxygen uptake (VO₂max). The model included the intrinsic capacity (IC) total score, the Fried frailty phenotype, age, and sex. Points represent standardized regression coefficients (β), and horizontal bars indicate 95% confidence intervals. Negative coefficients denote factors associated with lower VO₂max. Intrinsic capacity impairment remained independently associated with reduced aerobic capacity after adjustment for age, sex, and frailty status, highlighting the relationship between multidimensional functional decline and cardiorespiratory fitness in older adults.

Figure 2 illustrates the standardized regression coefficients from the final multivariable model, highlighting the independent contributions of intrinsic capacity, frailty status, age, and sex to absolute VO₂max.

## Discussion

This study provides novel insights into the interrelationships among frailty, intrinsic capacity, and objectively measured cardiorespiratory fitness in older adults attending a healthcare facility in India that has been under-represented in geriatric research. The prevalence of frailty in our cohort was high (73.1%), exceeding estimates reported in most community-based populations but consistent with studies conducted in healthcare-seeking older adults, where frailty prevalence ranges from 40-60% [27,28]. This difference likely reflects referral bias inherent to outpatient clinical populations, where individuals often present with greater multimorbidity compared with community cohorts. Frail participants were significantly older and demonstrated reduced body weight, skeletal muscle mass, and appendicular skeletal muscle index, findings that align with the recognized overlap between frailty and sarcopenia. These findings stem from a combination of anabolic resistance, hormonal decline, chronic low-grade inflammation, and neuromuscular degeneration, which collectively impair muscle contractility and metabolic efficiency, contributing to the progressive loss of physiological reserve that characterizes frailty [29]. Consistent with prior studies, frailty in our cohort was also associated with poorer functional performance, including reduced handgrip strength, slower gait speed, lower physical activity levels, and higher fall prevalence, reflecting the combined effects of neuromuscular weakness, impaired balance, reduced proprioception, and slower reflex responses that accompany aging and physical deconditioning [4,30]. 

Cardiorespiratory fitness was significantly impaired among frail participants. Frail individuals demonstrated lower absolute VO₂max, reduced oxygen pulse, and higher VE/VCO₂, suggesting impairments in both oxygen delivery and ventilatory efficiency. Maximal oxygen uptake represents the integrated capacity of the cardiovascular, pulmonary, hematologic, and skeletal muscle systems to transport and utilize oxygen during exercise. Reductions in VO₂max, therefore, reflect cumulative dysfunction across multiple physiological systems. Previous studies have reported similar associations between frailty and aerobic capacity, demonstrating moderate inverse correlations between frailty severity and VO₂max and have also shown higher VE/VCO₂ linked to poorer outcomes in frail participants [31,32]. Mechanistically, these impairments may arise from reduced maximal cardiac output, diminished skeletal muscle perfusion, and impaired mitochondrial oxidative capacity within aging skeletal muscle. Notably, in our cohort, VO₂max normalized to total body weight (VO₂max/kg) did not differ between frail and non-frail participants, whereas absolute VO₂max and VO₂max normalized to skeletal muscle mass (VO₂max/SMM) were both lower among frail individuals. One possible interpretation is that expressing VO₂max per kilogram of body weight can mask true reductions in aerobic capacity when body composition differs between groups. Frail older adults typically have less lean muscle and a relatively higher proportion of fat mass; because fat tissue consumes very little oxygen during exercise, dividing VO₂max by total body weight can make the frail and non-frail groups look more similar than they actually are [33]. This observation is consistent with a reduction in aerobic capacity per unit of metabolically active tissue rather than a simple effect of body size, although we interpret it cautiously, given the cross-sectional design and the use of bioelectrical impedance rather than reference methods for body-composition assessment. Within this physiological framework, intrinsic capacity provides a complementary perspective on aging-related decline.

In the present study, higher IC impairment scores were associated with poorer physical performance, reduced skeletal muscle mass, and lower absolute VO₂max, consistent with the concept that IC reflects early multisystem physiological decline. Among the five domains, locomotion and vitality showed the strongest associations with aerobic capacity-an expected pattern, since locomotion reflects skeletal muscle strength and neuromuscular coordination, and vitality encompasses nutritional status and energy metabolism, both of which directly shape exercise capacity [34,35]. The absence of association between IC and VO₂max normalized to body weight or skeletal muscle mass likely reflects the fact that IC itself incorporates functional and physical-performance domains that overlap with these normalized measures, whereas absolute VO₂max more directly captures integrated cardiopulmonary and metabolic reserve. Taken together, these findings support the study rationale that IC assessment, by capturing dimensions of physiological reserve not fully reflected in the frailty phenotype, may add practical value to frailty screening for identifying older adults with low aerobic capacity.

In the univariate analyses, several demographics, anthropometric, and functional variables were associated with maximal oxygen uptake. Age showed a strong inverse relationship with VO₂max, consistent with extensive physiological evidence demonstrating progressive reductions in aerobic capacity across the lifespan. Longitudinal studies have shown that VO₂max declines approximately 5-10% per decade after midlife, primarily due to reductions in maximal heart rate, stroke volume, and skeletal muscle oxidative capacity [9]. Skeletal muscle mass and appendicular skeletal muscle index were positively associated with VO₂max in our study, reinforcing the importance of muscle mass as a determinant of aerobic performance. Functional performance measures also demonstrated strong associations with VO₂max. Handgrip strength and gait speed were positively correlated with maximal oxygen uptake, reflecting the close relationship between neuromuscular function and cardiorespiratory fitness. These relationships highlight the interconnected nature of physical function and aerobic fitness in aging populations, as seen in previous studies [36].

Most individual comorbidities were not significantly associated with frailty, intrinsic capacity, or cardiorespiratory fitness in our cohort. This likely reflects a combination of factors: major chronic conditions such as diabetes and hypertension were distributed similarly across study groups, most participants were receiving regular outpatient care with generally well-controlled disease, and comorbidities were captured only as binary self-reported indicators, which under-represent disease severity and cumulative burden. Studies incorporating graded severity measures or composite comorbidity indices may be better positioned to detect associations with functional and aerobic reserve in this population.

In the multivariable analysis, intrinsic capacity remained independently associated with absolute VO₂max even after adjustment for age, sex, and frailty status. The persistence of IC as an independent predictor suggests that intrinsic capacity captures aspects of physiological decline not fully reflected by traditional demographic or phenotypic measures. Frailty, although strongly associated with VO₂max in univariate analyses, did not retain statistical significance after adjustment for IC. This finding supports the conceptual distinction between the two constructs. While frailty reflects a clinical phenotype of vulnerability characterized by weakness, exhaustion, and reduced activity, intrinsic capacity represents the underlying physiological reserves that determine an individual's resilience to stressors. It should nonetheless be acknowledged that the two constructs share operational overlap, most notably in gait speed (captured in the locomotion domain of IC and in the slowness criterion of the Fried phenotype) and in weight-loss or low-BMI items (captured in the vitality domain of IC and in the shrinking criterion of the Fried phenotype), and the persistence of IC in the multivariable model may therefore partly reflect its broader domain coverage.

Emerging longitudinal evidence suggests that declines in intrinsic capacity often precede the development of frailty, indicating that IC may function as an upstream determinant of vulnerability in aging populations [37]. An important methodological consideration in this study was the use of absolute VO₂max rather than VO₂max/kg measures as the primary outcome. Although VO₂max/kg is commonly used in exercise physiology studies, ratio scaling can introduce bias when body composition varies substantially across individuals. Older adults frequently exhibit sarcopenia accompanied by increases in fat mass, and because adipose tissue contributes minimally to oxygen consumption during exercise, normalizing VO₂ to total body weight may obscure meaningful differences in aerobic capacity.

Although the present study did not directly measure biological mediators, the observed associations between intrinsic capacity, frailty, and cardiorespiratory fitness are biologically plausible and can be interpreted within a broader conceptual framework of aging physiology. Prior work implicates chronic low-grade inflammation (inflammaging) and mitochondrial dysfunction as candidate mechanisms that together promote sarcopenia, reduce oxidative capacity in skeletal muscle, and thereby impair oxygen extraction and utilization during exertion [38]. Impairments within the vitality and locomotion domains of intrinsic capacity may represent early clinical manifestations of these processes, preceding measurable reductions in aerobic capacity and the eventual emergence of the frailty phenotype. Once established, reduced aerobic fitness may in turn accelerate further functional decline through reduced physical activity, muscle atrophy, and metabolic dysregulation, consistent with a reinforcing cycle described in previous longitudinal work. This hypothetical framework draws on prior mechanistic and longitudinal literature, and the associations observed in the present study are consistent with it.

Figure 1 illustrates the inverse association between IC impairment and absolute VO₂max in our cohort, and Figure 2 shows the independent contributions of age, sex, frailty, and IC in the multivariable model. The persistence of IC as an independent predictor after adjustment supports the conceptual distinction between the two constructs, while the substantial overlap in their components argues against treating this independence as evidence of construct primacy. From a clinical perspective, these findings suggest that combining a brief WHO-ICOPE intrinsic capacity screen with a Fried-phenotype frailty assessment may help identify older adults with low physiological reserve who might otherwise be missed by either tool used alone. This integrated approach is brief, low-cost, and feasible in resource-limited primary care settings common to low- and middle-income countries. Such case-finding is clinically meaningful only if matched with effective interventions: multicomponent exercise programs combining resistance training, aerobic conditioning, and balance work consistently improve muscle strength, mobility, and aerobic capacity in older adults [39], and combined exercise-and-nutrition interventions appear particularly beneficial in frail populations [40]. Prioritizing interventions that target the vitality and locomotion domains may offer the greatest yield given their observed links with aerobic capacity in this cohort. Prospective longitudinal studies are needed to confirm whether intrinsic capacity decline temporally precedes reductions in aerobic fitness and the emergence of frailty, and to evaluate whether integrated IC-plus-frailty screening improves outcomes when linked to targeted intervention.

This study has several strengths. First, it examined frailty, intrinsic capacity, and cardiorespiratory fitness simultaneously within a single analytical framework, enabling evaluation of the relationships between functional decline and physiological reserve in older adults. Second, cardiorespiratory fitness was measured using CPET, the reference standard for assessing maximal oxygen uptake, providing an objective evaluation. Third, intrinsic capacity was assessed using the WHO-ICOPE framework, allowing multidimensional evaluation. Fourth, the study contributes data from an under-represented South Asian geriatric population, addressing an important gap in the literature that has been dominated by Western cohorts. Finally, the primary analysis used a prespecified multivariable model adjusted for key demographic covariates, with explicit assessment of multicollinearity (all VIFs <1.31). Several limitations should be acknowledged. The cross-sectional design precludes inference about the temporal relationships between intrinsic capacity, aerobic fitness, and frailty. Participants were recruited from a tertiary outpatient geriatric clinic through convenience sampling, which may introduce referral and selection bias and limit generalizability to community populations. Comorbidities were recorded using binary self-reported indicators, which may not fully capture disease severity or cumulative burden, and several IC domains were assessed using Hindi-adapted but validated screening tools rather than comprehensive diagnostic assessments, potentially introducing measurement variability. The moderate sample size may have limited power to detect smaller associations. Importantly, frailty and intrinsic capacity share conceptual and operational overlap, most notably in gait speed and in weight-loss or low-BMI items, and despite the low statistical collinearity observed in our model, the persistence of IC as an independent predictor should not be interpreted as evidence of construct primacy over frailty. Finally, the biological mechanisms discussed in the Discussion were not directly measured in this cohort and are offered only as plausible explanatory contexts drawn from prior mechanistic literature, not as inferences from our data. Despite these limitations, the study provides informative insights into the associations between intrinsic capacity, frailty, and aerobic fitness in older adults.

## Conclusions

In this cross-sectional study of older Indian adults, greater intrinsic capacity impairment was independently associated with lower cardiorespiratory fitness after adjustment for age, sex, and frailty status. These findings indicate that intrinsic capacity captures aspects of physiological vulnerability not fully reflected by demographic or phenotypic frailty measures in this setting. Whether intrinsic capacity decline temporally precedes reductions in aerobic fitness and the emergence of frailty will require prospective longitudinal investigation. Integrating intrinsic capacity assessment with frailty screening and, where feasible, objective measures of aerobic fitness may assist in identifying older adults at risk of functional decline.
